# Drug Induced Sialorrhea and Microfluidic-Chip-Electrophoretic Analysis of Engorged Adult Female Tick Saliva of *Haemaphysalis longicornis* (Acari: Ixodidae)

**Published:** 2017-03-14

**Authors:** Mohammad Saiful Islam, Myung Jo You

**Affiliations:** 1 Laboratory of Veterinary Parasitology, College of Veterinary Medicine and Bio-safety Research Centre, Chonbuk National University, Jeonju, Republic of Korea; 2 Department of Medicine Surgery and Obstetrics, Faculty of Veterinary and Animal Science, Hajee Mohammad Danesh Science and Technology University, Dinajpur, Bangladesh

**Keywords:** *Haemaphysalis longicornis*, Induction, On-chip-electrophoresis, Salivation

## Abstract

**Background:**

The aim of the present study was to induce salivation in *Haemaphysalis* longicornis to increase saliva production and to characterize the collection of proteins present in the collected saliva using on-chip-electrophoresis.

**Methods:**

Saliva of adult female engorged *H. longicornis* was collected by treatment with 0.2% dopamine hydrochloride. All protein samples were characterized by SDS-PAGE electrophoresis using a microfluidic High Sensitivity Protein Assay 250 kit by 2100 Bioanalyzer (Agilent Technologies, USA) under non-reducing conditions.

**Results:**

The average salivary protein concentration was 0.169 μg/μl/tick and saliva secretion decreased with increased time of tick detachment from the host. Saliva secretion volume increased to 3.56 μl in the group of ticks with a body weight between 301–350 mg as compared to higher and lower body weight groups. On-chip-electrophoresis results show 13 distinct bands ranging from 9.9 to 294 kDa.

**Conclusion:**

Based on molecular weight, the putative salivary proteins are comprised of proline-rich proteins, triabin, apyrase members of the 12-kDa protein family, platelet inhibitors and anti-inflammatory proteins as tick saliva contains anti-inflammatory components.

## Introduction

“Ticks are ectoparasitic, blood-feeding arthropods that transmit pathogens to wild and domestic animals and rank second only to mosquitoes as vectors of human disease” (Zhang et al. 2011). *Haemaphysalis longicornis* is the most predominant species in Korea (>91.8%) and the prevalence of tick-borne pathogens by TaqMan PCR and species-specific PCR was frequent for some bacteria, including *Anaplasma platys*, *Ehrlichia Chaffeensis* and *Rickettsia* sp. in *H. longicornis* ticks for the most part (Kim et al. 2006). *Haemaphysalis longicorni*s also known vector of Q fever caused by *Rickettsia*, Russian Spring-Summer encephalitis caused by a virus, and theileriosis and babesiosis caused by protozoa species (Battsetseg et al. 2002). *H. longicornis* is also known to transmit Coxsackie-like virus (Hoogstraal 1973), Powassan encephalitis virus (Hoogstraal 1973), Khasan virus (Lavov et al. 1978), and *Borrelia* species (Hao et al. 2011). In addition to the pathogens they carry, ticks themselves causes severe toxic conditions including tick paralysis, various tick toxicoses, irritation, and tick bite allergies. Losses due to tick infestations can be considerable. In 1974, Australian husbandry losses due to the cattle tick (*Boophilus microplus*) were estimated at 62 million USD (Springell 1983) and Brazil economically loses around 2 billion USD per year (Grisi et al. 2002).

During tick infestation, the tick secretes bioactive substances that modify the host’s physiological and immunological reactions. Macromolecules present in the saliva consist of lipids and proteins, which alter physiological changes at the feeding site thereby affecting pathogen transmission. Tick salivary glands produce a large proportion of these substances that are secreted into the host during blood feeding. Based on proteomic studies, many tick salivary proteins with specific functions have been identified (Hovius et al. 2008). Tick salivary immunosuppressive proteins interfere with the host’s innate and adaptive immune responses, which may result in more efficient transmission of several tick-borne pathogens (Zhang et al. 2011).

The study of tick saliva is important to understand tick biology as tick saliva plays a special physiological role in pathogen transmission. Due to the importance of tick saliva in inflammation, pathogen transmission, immunity and hemostasis, the isolation and characterization of saliva molecules responsible for these effects is the most important to understanding host-pathogen immune reactions in vector borne diseases. However, the amount of protein in tick saliva is very limited and the isolation of protein components from the saliva is a big challenge. To overcome this challenge, researchers have studied salivary glands in lieu of saliva for the molecular characterization of protein compounds. Salivary proteins are classified as concealed or exposed. By default, studies with salivary glands lack assessment of exposed proteins.

The purpose of the present study was to induce salivation in *H. longicornis* to increase saliva production and to characterize the collection of proteins present in the collected saliva using on-chip-electrophoresis. Results of this may help researchers to identify tick proteins as potential candidates for further studies aimed to develop novel tick control strategies.

## Materials and Methods

### Ticks

The Jeju strain of the hard tick *H. longicornis*, collected form Jeju Island in Korea has been maintained on rabbits in our laboratory since 2003. Engorged female ticks were used for the analysis of salivary proteins. At least 150 clean *H. longicornis* adults were placed on each ear of 4–5 week old female New Zealand white rabbits (Samtako, Korea) and the ticks were allowed to attach and feed to repletion. Engorged female ticks dropped from the rabbit ear were assessed for body weight and kept in plastic tubes at 27 °C under humid conditions.

The Chonbuk Animal Care and Use Committee approved all rabbit studies and the protocols are in agreement with the ethical principles for animal research.

### Saliva collection

**Fig. 1.**
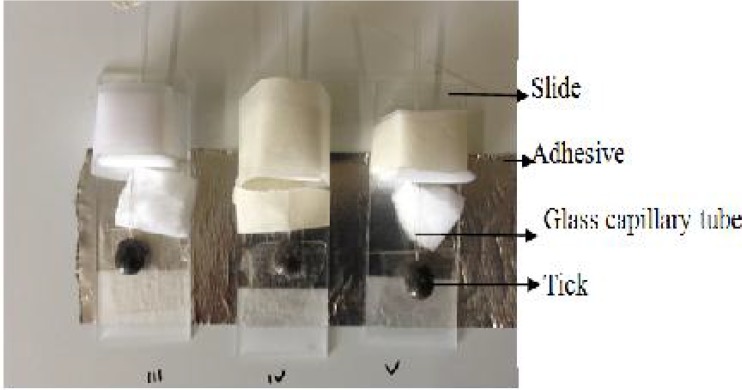
Collection of saliva from engorged adult female *Haemaphysalis longicornis* after injecting with 0.2% dopamine

Dropped engorged female *H. longicornis* were carefully collected and sterilized by wash in 70% ethanol for 5 min. The ticks were then rinsed in at least three changes of sterile distilled water. The ticks were gently ventrally attached to a slide using double-sided bonding tape for tick immobilization and observation under a stereo zoom microscope (Nikon SMZ-U, Japan). A 3–5 μl aliquot of 200 mg/5 ml dopamine hydrochloride solution (Hunguhsu Pharma, Korea) in 0.2% phosphate-buffered saline, pH 7.4, was introduced into the posterior to fourth coxae in the region of epimeral and anal plates each tick using a micro-fine specially made glass needle (Fig. 1). Tick saliva was harvested from the tick mouthparts using a glass microtube and stored at −80 °C until further use. If the tick failed to produce saliva by 5 min post-injection, the tick was re-injected. If the tick failed again within 5 min of the second injection, it was discarded from the study. Saliva was collected from 120 out of 150 ticks. Total protein concentration of the saliva was determined using a Bio-Rad Protein assay based on the method of Bradford (Bio-Rad Laboratories, Hercules, CA, USA).

### On-chip-electrophoresis

All protein samples were characterized by SDS-PAGE electrophoresis using a microfluidic High Sensitivity Protein Assay 250 kit and a 2100 Bioanalyzer (Agilent Technologies, USA) under non-reducing conditions. All proteins were observed as single bands in the electropherograms. The protocol for on-chip-electrophoresis was followed per the manufacturer’s instructions with slight modification to improve results with diluted labelled protein. Briefly, six different dilution samples were loaded onto six chips (Fig. 4A, B). Tick saliva was divided as three groups based on dilution as sample 1, no dilution, sample 2, 1:10 dilution with TE buffer and sample 3, 1:100 dilutions with TE buffer. Each of three sample were duplicated to load in six different well of Agilent high sensitivity protein 250 chip and analyzed.

### Recording data and analysis

Twenty ticks were divided into five groups based on body weight at less than 250 mg, 251–300 mg, 301–350 mg, 351–400 mg, and more than 400 mg. On the day of detachment, the ticks were treated with dopamine as described for saliva collection and the data were recorded to determine the relationship between body weight and salivation. Saliva was collected for five consecutive days. The data were subjected to ANOVA with SAS (Ver.8.x). Duncan’s multiple range tests was used to determine significant differences among the various durations or ticks body weight.

## Results

**Fig. 2.**
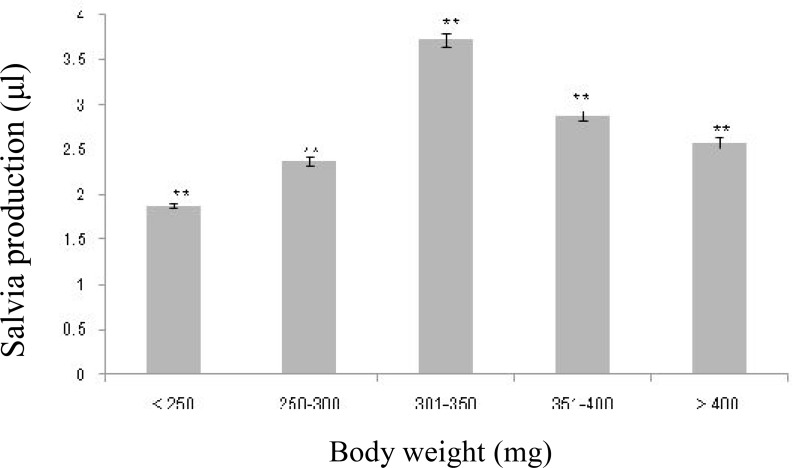
Body weight vs. saliva production from engorged adult female *Haemaphysalis longicornis.* Ticks are collected after spontaneous detachment between 5 to 7 days. Values presented are the mean ±SD. **identifies significance between two groups (P< 0.001)

**Fig. 3.**
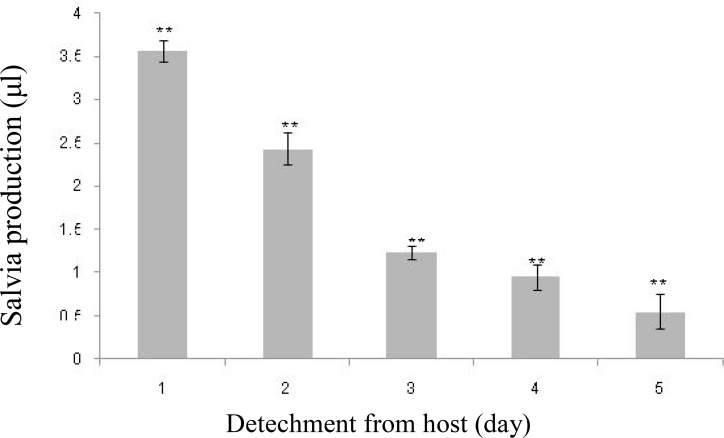
Saliva production vs. detachment time (day) from engorged adult female *Haemaphysalis longicornis* three-host tick Values presented are the mean ±SD. **identifies significance between two groups (P< 0.001).

**Fig. 4.**
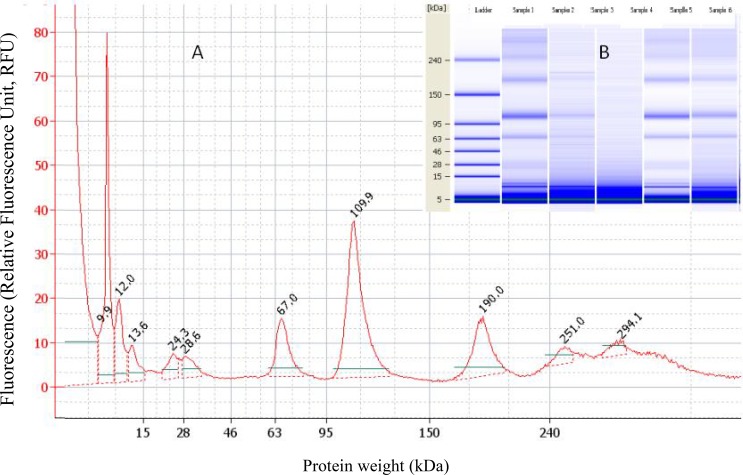
On-chip-electrophoresis. A. Gel view. B. Electropherogram with the peak relative to the protein bands shown in column 4 (RFU, relative fluorescence units)

The measured protein concentration was found to be 0.169 μg/μl. This method of saliva collection used dopamine to stimulate salivation and yielded over 1.74 μl of saliva per adult engorged female tick. The maximum saliva production was found from the ticks that were treated immediately after detachment from the tick. Saliva production decreased with an increase of time after detachment. At day 1 post detachment, the average saliva production was 3.56 μl saliva per adult engorged female tick while at day 5 the average production was only 0.55 μl per adult engorged female tick (Fig. 3). In addition, salivation significantly (P< 0.001) increased with an increase in body weight from less than 251–350 mg body weight group of engorged ticks. The 301–350 mg tick group body weight produced the highest volume of saliva at an average of 3.7 μl/tick. However, the ticks that weighed more than that showed a decrease in saliva volume as ticks of more than 400 mg body weight produced on an average 2.57 μl of saliva per engorged female (Fig. 2). A total of 13 protein bands were identified based on the on-chip-electrophoresis analysis. In the electrophoretogram, the strong signals were at 109 kDa (948 pg/μl), 9.9 kDa (900 pg/μl), 190 kDa (332 pg/μl), 12 kDa (327 pg/μl), 67 kDa (311 pg/μl), and 10.3 kDa (213 pg/μl). Other protein bands identified were at 13.6, 24.3, 28.6, 251.0, 294.1, 108.2, 68.1, and 110.4 kDa (Fig. 4 and Table 1).

**Table 1.** Protein bands and their relative concentration found from saliva of adult female engorged *Haemaphysalis Longicornis*

**No**	**Size [kDa]**	**Relative concentratin (pg/μl)**
**1**	9.9	900.1
**2**	10.6	68.4
**3**	12.0	327.5
**4**	13.6	166.2
**5**	24.3	120.5
**6**	28.6	119.7
**7**	67.0	311.6
**8**	108.2	31.7
**9**	109.9	948.2
**10**	110.4	49.9
**11**	190.0	332.7
**12**	251.0	68.8
**13**	294.1	56.2

## Discussion

Tick hemolymph, salivary glands, and saliva are important tools for the study of tick-borne pathogen transmission, adaptation, growth, and multiplication in both the tick and the host (Labuda and Nuttall 2004, Horka et al. 2009, Socolovschi et al. 2009). *Haemaphysalis longicornis* adult tick length (unengorged) is approximately 2.65 mm (2.7–3.4 mm) with a breadth of 1.8 mm (1.4–2.0 mm). Although body weight increases after engorgement, there is no detectable change to the size of the mouthpart. For this reason, researchers typically use salivary glands to collect protein extracts instead of saliva for proteomic analysis of *H. longicornis* (Lvov et al. 1978, Mulenga et al. 1999, Wang et al. 2001 Oliveira et al. 2011, Jinlin et al. 2013, Wikel 2013). When collecting saliva, it is critical to inject the tick so the salivary glands are not ruptured or lost in the tick remains. Saliva contains additional proteins originating from the salivary gland cells that are not present in tick salivary gland extract itself. Many chemical stimulatory techniques have been used for saliva collection, the most common of which are dopamine and pilocarpine (Kramer et al. 2008, Patton et al. 2012, Oliveira et al. 2013).

Although the hemolymph and other tick fluids rapidly degrade dopamine, it has selective organ action. Other stimulatory agents such as pilocarpine have been explored for saliva collection; however, all were shown to affect salivary composition as their action has affects on multiple cell types (Ribeiro et al. 2004). In this study, we used dopamine to reduce the chance of changing saliva composition. For the first time, we successfully collected saliva from *H. longicornis* and characterized that collected saliva via on-chip-electrophoresis analysis. Our collected saliva induced by dopamine was ash in color and produced a precipitate following centrifugation at 10,000 × *g* for 1 min, which is similar to the collected saliva by Oliveira et al. (2013). The color change may be due to dopamine itself, most likely because dopamine triggers the phenoloxidase pathway.

The protein concentration of these saliva samples was on an average 0.169 μg/μl for engorged *H. longicornis* females. The concentration of saliva induced by dopamine found in *Ornithodoros moubata* ticks was 0.33 μg/μl for females (Wang et al. 2001) and 0.184 μg/μl for *Rhipicephalus sanguineus* ticks (Oliveira et al. 2011).Our saliva concentration results resemble the protein concentrations found for *R. sanguineus*, which is predictable as these parasites are almost identical physiologically.

Saliva collection by our method yielded approximately 1.74 μl per tick with an average high production on day 1 at 3.56 μl and an average low production at 0.55 μl. Lucas et al. (2014) found an average of 0.8 μl saliva per fully engorged *R. microplus*. Saliva production decreased in engorged ticks with an increase of time to treat with dopamine. This may be due to degeneration of the salivary gland after detachment from the host. In ticks, the salivary gland is also responsible for hydrodynamic equilibrium (Benoit and Denlinger 2010) and before detachment from the host ticks excretes more water. Due to this, our collected volume of saliva was higher compared to partially fed females as they were found to secrete 0.1 μl saliva per tick (Lucas et al. 2014).

On-chip-electrophoresis is a good approach for saliva characterization. The commercial Agilent High Sensitivity Protein 250 kit and Bioanalyzer 2100 (Agilent Technologies, USA) system were used for saliva protein determination. This technique is a combination of chip microfluid separation technology and the fluorescent detection of proteins. It automatically performs all the steps of gel-based electrophoresis including sample separation, staining, imaging, band detection, and data analysis. In our electrophoresis results, intense bands were identified at 9.9, 10.3, 10.6, 12, 67, 109, and 190 kDa. Proline-rich proteins form a major fraction of salivary proteins (approximately 20–30% of total), and the molecular weight of proline-rich proteins (acidic and basic) is usually between 10–40 kDa while large glycosylated proline-rich proteins range from 60–70 kDa (Schwartz et al. 1995). The major sources of salivary proline-rich proteins (PRPs) are the salivary glands (Bishop et al. 2002).

A 9.9-kDa salivary protein was found in *T. infestans* (Charneau et al. 2007) and a 10.6 kDa protein was isolated from *T. infestans* as a triabin protein. Triabin belongs to the family of lipocalins that are extracellular transport proteins and have platelet inhibitor function (Andersen et al. 2005). The 67-kDa protein corresponds to tick salivary apyrases known to inhibit ADP and collagen. These properties indicate that apyrases could play an important role during tick feeding (Mans et al. 1998). The 12-kDa protein is another characteristic protein family exclusive to both hard and soft ticks that was previously found only in *Ixodes scapularis*, *O. coriaceus*, and *O. moubata* (Francischetti et al. 2009). The proteins that resemble the 10.6, 12, and 67 kDa proteins are likely platelet inhibitors and anti-inflammatory proteins, as tick saliva is known to contain anti-inflammatory components (Chmelar et al. 2011, Fontaine et al. 2011). When Oliveira et al. (2013) treated *R. sanguineus* with pilocarpine, they found rabbit host proteins in the collected saliva. None of our salivary proteins collected via dopamine treatment matched any rabbit host protein sequences. Even still, saliva collected by the dopamine technique has some limitations as only 0.2% dopamine was used and further dose dependent studies need to be conducted to optimize saliva production. An additional problem is the color change of the saliva, most likely due to activation of the phenoloxidase pathway, which could be verified by adding phenoloxidase inhibitors such as DTT or EDTA to the saliva. Lastly, proteomic studies are required to characterize further the protein bands identified in this study.

## Conclusion

As most of the protein bands identified in this study do resemble tick salivary proteins, this method could be a useful tool for the primary identification key of *H. longicornis* antigens for vaccine development.
